# The Foot That Broke Both Hips: A Case Report and Literature Review of Tumor-Induced Osteomalacia

**DOI:** 10.1155/2017/3191673

**Published:** 2017-09-14

**Authors:** Sara Beygi, Alfred Denio, Tarun S. Sharma

**Affiliations:** ^1^Internal Medicine Department, Allegheny Health Network, Pittsburgh, PA, USA; ^2^Rheumatology Department, Geisinger Medical Center, Danville, PA, USA; ^3^Rheumatology Department, Allegheny Health Network, Pittsburgh, PA, USA

## Abstract

Tumor-induced osteomalacia (TIO) is a rare paraneoplastic syndrome characterized by hypophosphatemia and clinical symptoms of osteomalacia. Only discussed as case reports, there is still limited knowledge of this condition as a potentially curable cause of osteomalacia among clinicians and pathologists. In this article, we present a case of tumor-induced osteomalacia in a 59-year-old gentleman followed by an up-to-date review of the existing literature on TIO.

## 1. Introduction

Tumor-induced osteomalacia (TIO) or oncogenic osteomalacia is an underrecognized paraneoplastic syndrome presenting with hypophosphatemia and typical manifestations of osteomalacia [1]. The clinical syndrome of osteomalacia and associated biochemical abnormalities including low phosphate and 1,25-dihydroxyvitamin D levels are blamed on the production of phosphate-regulating substances, most notably fibroblast growth factor 23 (FGF23), by tumor cells [2, 3]. Since the FGF23-producing tumors tend to be small and hard to detect on physical exam, symptoms of osteomalacia, including bone pain and muscle weakness, sometimes precede the diagnosis of the tumor by years. The fact that surgical resection of the tumor is usually curative highlights the importance of raising awareness of this condition among clinicians and pathologists.

In this article, we present a case of TIO followed by a review of the current literature.

## 2. Case Presentation

A 59-year-old gentleman was hospitalized in May 2013 for elective repair of a stress fracture of the medial midshaft of the right femur. Orthopedic surgeons planned to perform an elective surgery. A routine rheumatology consult as a part of the fracture liaison service was requested. Upon further questioning, he admitted to a minor fall while carrying a heating unit up a flight of stairs a few months prior to admission. Since then, he had persistent discomfort in the right thigh and was complaining of difficulty walking, loss of balance, and leg “giving out on him” for a few months. His past medical history was only remarkable for hypertension and cervical spinal stenosis, and his social/family history was noncontributory.

Physical examination revealed a man of normal stature with normal-appearing limbs. He had tenderness to palpation of the right medial midthigh region. In addition, some mild tenderness in the left midthigh was noted. The remainder of the exam including testicles, sclera, and skin elasticity was unremarkable.

**Figure 1:**
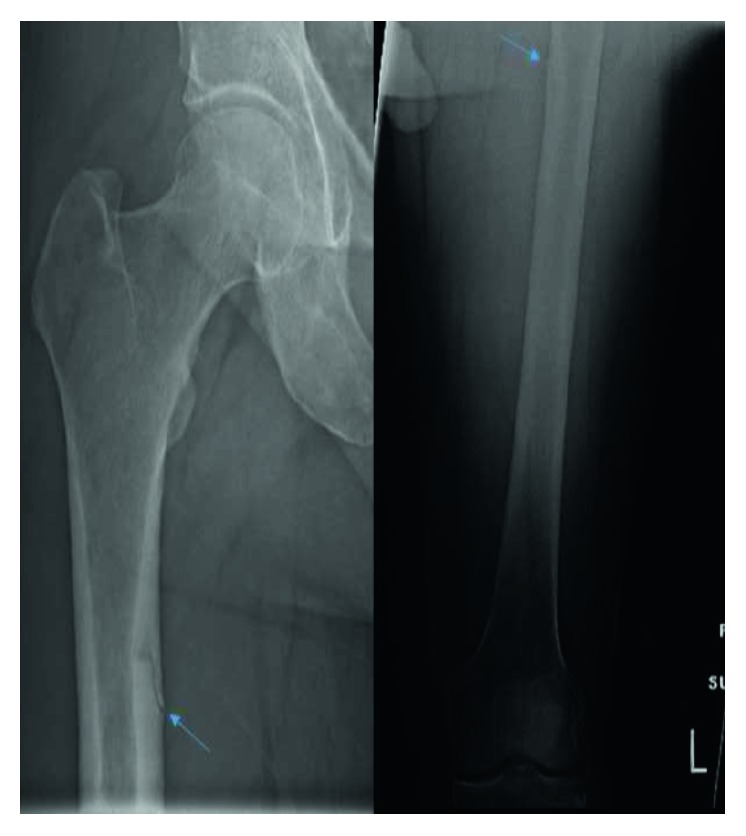
Radiographic image of both femurs with arrows pointing towards the fracture lines (the right femur on the left side and the left femur on the right side).

Preoperative workup included an AP pelvis X-ray which revealed a left subtrochanteric femur stress fracture in addition to the known right femur fracture (Figure 1()).

An intramedullary device was placed bilaterally.

Laboratory parameters obtained prior to surgery are as follows:

- Calcium: 9.4 mg/dL (*n*: 8.3–10.5)
- Alkaline phosphatase: 135 U/L (*n*: 0–153)
- 25-Hydroxyvitamin D level: 22 ng/mL (*n*: 30–100)
- 24-hour urine calcium: 0.223 g/24 hours (*n*: 0.05–0.3)
- TSH: 2.24 uIU/mL (*n*: 0.27–4.2)
- Creatinine: 1.0 mg/dL (*n*: 0.7–1.5)
- iPTH: 50 pg/mL (*n*: 10–65)
- *Phosphate: 1.5 mg/dL* (*n*: 2.5–4.8)
- 24-hour urine phosphate: 0.821 g/24 hours (*n*: 0.3–1)
- *Plasma fibroblast growth factor 23 (FGF23): 211 RU/mL* (*n* < 180)

**Figure 2:**
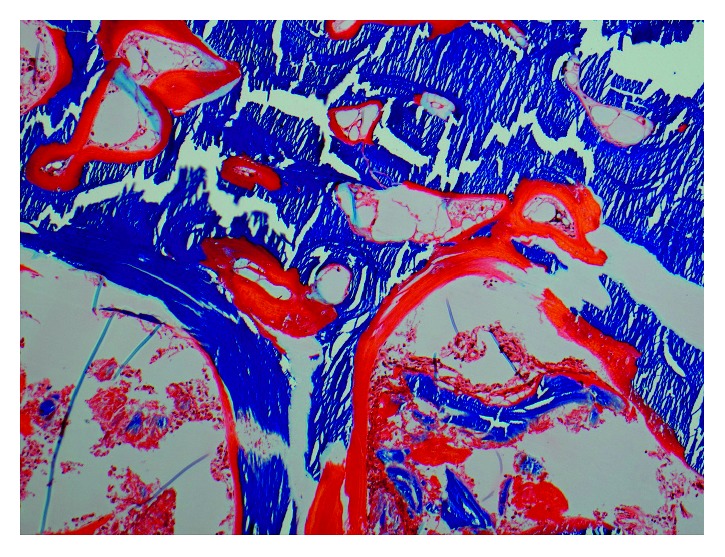
Plastic-embedded trichrome-stained pictures of the bone with 20x magnification. The trichrome staining shows wide seams of unmineralized osteoid (red) that covers virtually all trabecular surfaces, suggesting a severe mineralization defect.

**Figure 3:**
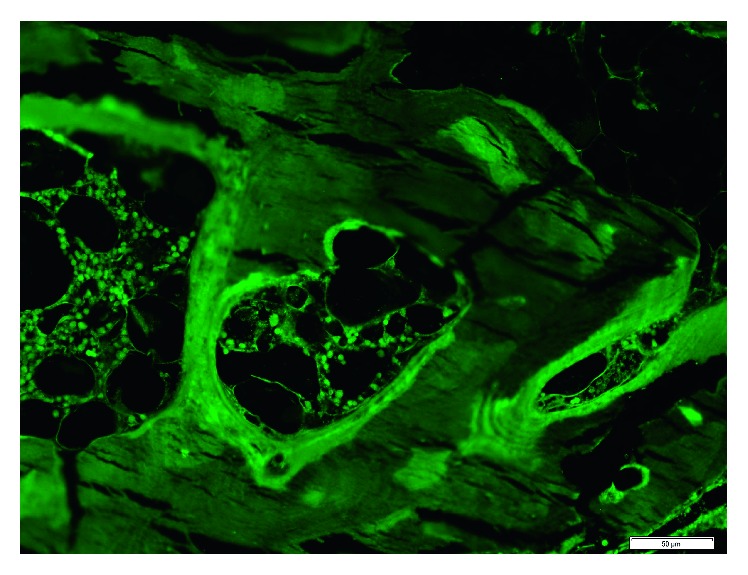
Fluorescence microscopic pictures of the tetracycline-labeled bone with 20x magnification. A severe mineralization defect is confirmed by an unstained section of the bone under fluorescence microscopy looking for tetracycline labeling which reveals faint fluorescence with a blurred pattern and no evidence of the typical double labeling of the bone matrix.

An iliac crest bone biopsy with trichrome staining (Figure 2()) and tetracycline labeling (Figure 3()) revealed marked osteomalacia and a severe mineralization defect.

**Figure 4:**
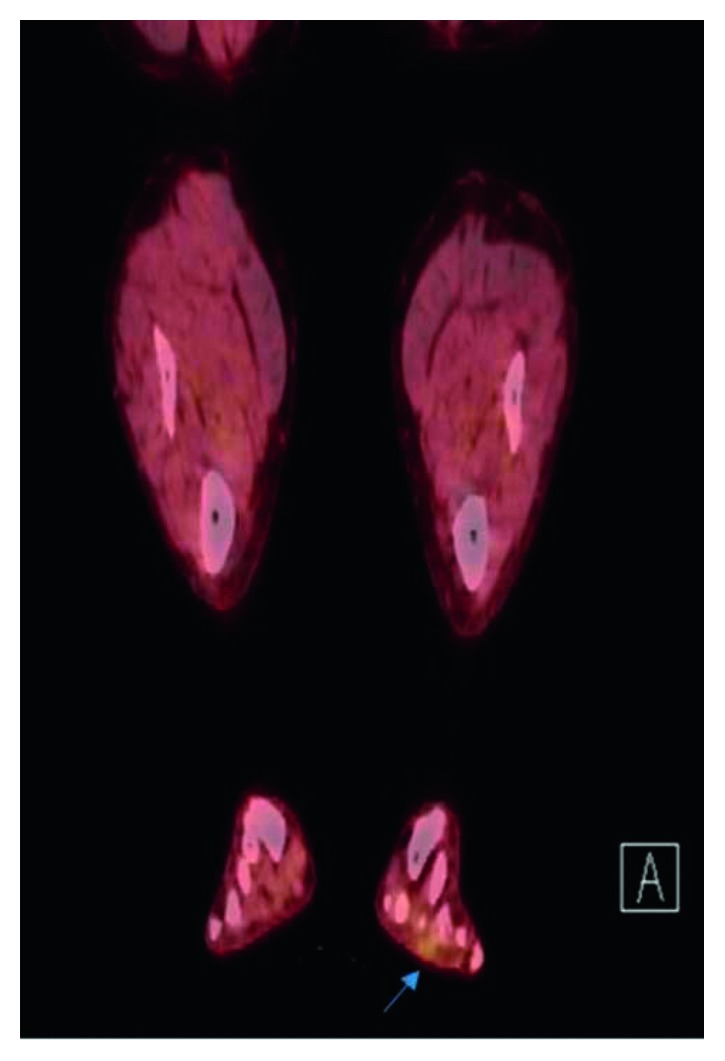
Positron emission tomographic image of the lower extremities showcasing a fluorodeoxyglucose (FDG) avid spot on the plantar surface of the left foot (arrow).

A whole body PET/CT revealed uptake within a soft tissue nodule in the plantar surface of the left foot, at the level of the 2nd and 3rd metatarsophalangeal joints (Figure 4()).

**Figure 5:**
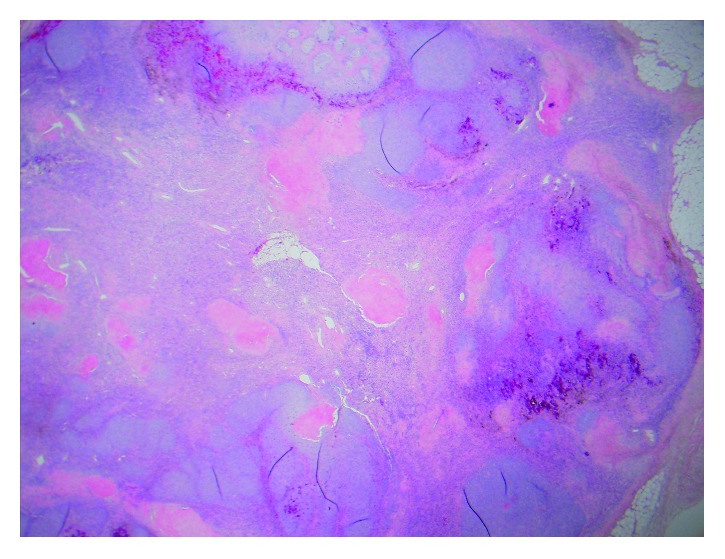
Low-power microscopic image (2x) showing a well-delineated tumor consisting of variegated mesenchymal components rich in small vessels with focal myxoid stroma.

Excisional biopsy of the left plantar foot nodule revealed a benign mesenchymal tumor consisting of small vessels admixed with fibrocartilage foci (Figures 5()–7()).

The tumor was resected, and the patient's FGF23 and phosphate levels returned to normal soon after the surgery (phosphate: 4.6 mg/dL day 11 post-op, FGF23: 76 RU/mL 4 months post-op). The patient has been following up with an orthopedic oncologist every 6 months. The FGF23 and phosphate values continue to be within normal limits during the follow-up period, and the patient remains asymptomatic with no indications of recurrence (phosphate: 2.8 mg/dL, FGF23: 83 RU/mL at 14-month follow-up).

## 3. Literature Review Method

A PubMed search was performed on January 1, 2017, using the following search strategy:

((((((oncogenic osteomalacia[Title/Abstract]) OR oncogenic hypophosphatemia[Title/Abstract]) OR tumor induced osteomalacia[Title/Abstract]) OR tumor induced hypophosphatemia[Title/Abstract]) OR tumor-induced hypophosphatemia[Title/Abstract]) OR tumor-induced osteomalacia[Title/Abstract]) OR phosphaturic mesenchymal tumor[Title/Abstract].

A total of 490 articles were retrieved and reviewed.

## 4. Background

For the first time in 1947, McCance reported a 15-year-old patient with weakness, gait disturbances, and low phosphate level whose symptoms completely resolved after resection of a tumor found in her femur [4]. However, her condition was inaccurately attributed to vitamin D resistance. As it is inferred from the literature, back in those days vitamin D resistance was believed to be the mechanism of what was eventually recognized as FGF23-induced phosphate-wasting disorder [5]. The first person who blamed the disease on the production of a “rachitogenic” substance by tumor cells was Prader [6]. He described severe rickets in an 11-year-old girl who was found to have a giant cell granuloma of the rib. Complete resolution of her rickets was achieved following resection of the tumor. Prader clearly delineated the association between resolution of the symptoms and tumor resection and proposed that the granuloma might have released a rachitogenic mediator. Since recognition of the condition, around 350 cases have been reported in the literature, most of them being published over the last 10 years.

It was repeatedly noted by different experts that TIO-associated tumors share distinctive morphological features, favoring an unidentified histopathological entity [7]. The term “phosphaturic mesenchymal tumor” (PMT) was introduced by Weidner and Santa Cruz [8], and PMT was eventually added to the 2013 WHO classification of tumors of the soft tissue and bone [9].

## 5. Clinical Characteristics

Tumor-induced osteomalacia typically occurs in adults, with equal gender distribution [10]. Patients usually present with progressive musculoskeletal symptoms including pain, proximal muscle weakness, and fractures. Due to general unawareness of this condition, there is usually a significant lag between the onset of symptoms and diagnosis. Therefore, it is not uncommon for patients to present with multiple fractures occasionally leading to height loss and a debilitated state [11].

The classic biochemical profile is characterized by normal calcium and parathormone levels, low or inappropriately normal 1,25-dihydroxyvitamin D levels, and elevated alkaline phosphatase levels [12].

Differentiating TIO from acquired and hereditary disorders that could present with hypophosphatemic osteomalacia would be of paramount importance. The acquired causes include nutritional deficiencies of vitamin D or phosphate as well as renal tubular abnormalities due to a variety of causes such as burns, heavy metal exposure, certain medications, and paraproteinemia [1, 13]. The inherited causes of hypophosphatemic osteomalacia include X-linked hypophosphatemic rickets (XLH), autosomal dominant hypophosphatemic rickets (ADHR), autosomal recessive hypophosphatemic rickets (ARHR), and hereditary hypophosphatemic rickets with hypercalciuria (HHRH) which are biochemical equivalents of TIO with high or inappropriately normal levels of FGF23 [1, 13].

Phosphaturic mesenchymal tumors tend to be small and not easily detectable on physical exam [10]. In fact, it is the osteomalacia and associated metabolic abnormalities which usually raise suspicion for an underlying tumor. TIO has been reported in association with other types of tumors as well, most notably small cell carcinoma and neurofibromatosis [14], although this is usually a feature of PMTs. However, unlike PMTs, in the case of TIO associated with other tumors, the primary tumor is usually known at the time of presentation. This type of presentation is sometimes referred to as secondary TIO [13]. Interestingly, few cases of TIO secondary to colon adenocarcinoma and ovarian cancer have also been reported [15, 16].

Reviewing the reported cases of TIO to date reveals that 50–55% of the tumors have been reported to arise from soft tissues and 40–45% in bones. Thigh and femur tend to be the most common anatomical sites (22.7%), followed by the craniofacial region (20.7%), ankle and foot (8.8%), pelvis (8.2%), tibia and fibula (6.5%), and arms (6.5%) [17]. In the head and neck region, three-fourths of the cases were noted in extraoral sites, with paranasal sinuses being the most common location [10]. There has not been a single report of the retroperitoneum or parenchymal organ as the primary site of the tumor [18].

## 6. Histopathological Features

The classic histological appearance of phosphaturic mesenchymal tumors consists of a background of spindle- to stellate-shaped neoplastic cells with a “smudgy” appearance which are generally normochromic with small nuclei and indistinct nucleoli [1]. The nuclear grade and mitotic activity are characteristically low. The cells are typically nested within a myxochondroid matrix with so-called “grungy” calcifications resembling the cartilage or osteoid tissue. Osteoclast-appearing giant cells are a common finding while mature lamellar bone may also be noted. A prominent feature of these tumors is an elaborate microvascular network with vessels of various sizes and patterns resembling a “staghorn” [13]. Microcytic changes have also been frequently described. Lately, immunostaining with D2-40, an antibody against a lymphatic marker, has proven to be positive in analysis of the fluids from microcysts, suggesting a partial differentiation toward lymphatic endothelial cells [19]. Initially, four different histological variants of PMTs were described: mixed connective tissue variant (PMTMCT), osteoblastoma-like variant, nonossifying fibroma-like variant, and ossifying fibroma-like variant [20]. However, later on, Folpe et al. [21] demonstrated that almost all previously identified mesenchymal tumors would fall under the single category of PMTMCT, debating the previous notion of 4 different histological subtypes. Of all the immunohistochemical markers investigated so far, only 2 have been tested positive, smooth muscle actin and FGF23, in 10 and 70% of the cases, respectively [21]. Of note, only proliferating cells within the tumor were noted to stain for FGF23 [21]. Somatostatin receptors have also been consistently detected in TIO tumors [22]. Therefore, it has been suggested that a positive staining for both FGF23 and somatostatin receptors would be a sensitive test for PMTs, though probably not specific enough [22].

PMTMCT also manifests ultrastructural features of neuroendocrine tumors [23]. However, typical immunohistochemical markers of neurosecretory tumors including S-100, neuron-specific enolase, chromogranin, and synaptophysin have not been detected [13]. Perhaps, vimentin is the only neurosecretory marker that has consistently been noted positive [24]. Interestingly, there are reported instances of tumors with histopathological features consistent with PMTMCT, though not associated with the clinical syndrome of osteomalacia. These tumors are sometimes classified as nonphosphaturic variant [21, 25].

Although characteristically benign, malignant behavior and metastases have occasionally been reported [26, 27]. Similarly, there have been rare instances of locally aggressive lesions as well as multifocal lesions [18]. It is worth noting that, even in the case of metastasizing tumors, the histopathological appearance of the tumor tends to be benign. On the other hand, microscopic infiltration of the surrounding soft tissue is often noted in the margins of an otherwise benign-appearing lesion [18].

## 7. Pathogenesis

Overexpression of phosphate-regulating factors has been consistently implicated in the pathogenesis of tumor-induced osteomalacia. Among all, FGF23 has perhaps the most established role. FGF23 was identified as the culprit phosphaturic substance after mutations in FGF23 gene were recognized in the pathophysiology of ADHR [28]. FGF23 is physiologically produced by normal bone cells [29] and, aside from its phosphaturic action, is thought to act directly on the bone and suppress differentiation of osteoblasts [30]. There is some evidence that the phosphaturic effect of FGF23 is somewhat PTH dependent. The notion is supported by the observation that in the presence of low to undetectable PTH levels, as occurs in hypoparathyroidism, FGF23 does not lead to hypophosphatemia [31].

To date, several other phosphate-regulating factors including matrix extracellular phosphoglycoprotein (MEPE) [30], secreted frizzled related protein-4 (sFRP-4) [32], and dentin matrix protein 1 (DMP1) [33] have been identified. However, their precise pathogenicity remains to be elucidated. Of note, despite a strong expression of messenger ribonucleic acid (mRNA) of the aforementioned factors by tumor cells, elevated plasma levels have not yet been reported in the setting of TIO. More recently, Lee et al. [34] identified a fibronectin 1–fibroblast growth factor receptor 1, FN1–FGFR1, fusion in 42% of the studied phosphaturic mesenchymal tumors (21/50). Additionally, a novel FN1–FGF1 fusion gene was reported in 6% of the cases (3/50) [34]. Lately, a case of TIO with elevated venous levels of both FGF7 and FGF23 has been reported, suggesting a possible phosphaturic role for FGF7 as well [35]. The phosphate-regulating factors act primarily on the proximal renal tubules, causing redistribution of sodium phosphate cotransporters and therefore impairing reabsorption of phosphate [36]. Moreover, as a secondary mechanism, FGF23 and sFRP-4 decrease vitamin D levels presumably through downregulation of 1α-hydroxylase and upregulation of 24-hydroxylase [32, 36].

## 8. Diagnosis

Diagnosis of TIO should always be suspected in case of hypophosphatemia and excessive phosphaturia. Some of the notable causes of hypophosphatemia are listed in Table 1. A thorough history including family history of genetic phosphate abnormalities along with a complete biochemical testing is recommended as the next step (Table 2). A typical patient with TIO manifests low serum phosphate, normal to low calcium, normal PTH, normal 25-hydroxyvitamin D, normal to low 1,25-dihydroxyvitamin D, elevated bone alkaline phosphatase, and high FGF23 levels.

After a biochemical profile supports the diagnosis of TIO, an exhaustive physical exam is warranted to locate the tumor. However, as discussed earlier, these tumors tend to be obscure and typically very hard to detect on physical exam, therefore prompting the use of functional and anatomical imaging modalities. On the other hand, precise localization of the tumor is of paramount importance since resection of the tumor is usually curative [1]. Overall, F-18 fluorodeoxyglucose positron emission tomography, coupled with computed tomography (FDG-PET/CT), is thought to be the most sensitive method for localizing TIO tumors [37]. However, poor specificity limits the value of this modality. Indium-111 octreotide scintigraphy is another useful functional modality which takes advantage of the high affinity of indium octreotide for somatostatin receptors, especially subtype 2 which is most frequently detected in PMTMCT [38]. One important consideration would be to ensure coverage of the entire body as opposed to the standard frame of PET/CT and octreotide scanning which excludes distal extremities as well as parts of the head [39]. A sensitivity of 60–80% has been suggested for octreotide scintigraphy [40] and PET/CT [37] for detection of the PMTs in small case series. Somatostatin receptor PET tracers, Ga68-DOTANOC [41] and Ga68-DOTATATE [42], have emerged as potential agents for localization of PMTs. Labeling a modified octreotide molecule, with high affinity for both somatostatin receptors 2 and 5 [43] with Ga68, a positron emitter, allows for an admixture of PET sensitivity and octreotide specificity. Recent reports have supported superiority of Ga68-DOTATATE to FDG-PET/CT in the diagnosis of TIO [42], as well as in the case of nonrevealing prior indium-111 octreotide scan [44].

Once a suspicious tumor is identified through functional studies, anatomical imaging needs to be pursued to confirm the location and further characterize the lesion. Magnetic resonance imaging (MRI) is the most frequently utilized anatomical modality [45]. The most typical radiographic appearance of PMTs is a well-defined lytic lesion on computed tomography (CT) and a T2 hyperintense and enhancing lesion on MRI. However, occasionally lesions expanding beyond the bone, resembling a primary bone malignancy, have also been noted [18].

Overall, different diagnostic approaches have been recommended in the current literature. Some authors advocate a multistep approach starting with functional modalities, most notably PET/CT [13]; however, other experts prefer to use MRI as the initial step [32]. If imaging studies fail to localize a suspected lesion, venous sampling could be the next step. However, blind venous sampling in the absence of a previously identified suspicious lesion on functional or anatomical imaging has not led to promising results [46]. Aspiration of the suspected lesion and measuring FGF23 levels in the aspirate along with microscopic evaluation of the floating cells may be of diagnostic value [47]. Finally, if tumor localization efforts are unsuccessful, serial imaging with 1-2-year interval may be a reasonable course of action. Once the tumor is localized and resected, microscopic evaluation of the lesion allows for an accurate diagnosis of the PMT. FGF23 expression can be confirmed by the reverse transcription polymerase chain reaction (RT-PCR) technique on the tissue and also by anti-FGF23 antibodies which are still not widely available [48]. It is worth noting that the exquisite sensitivity of the molecular method in detection of FGF23 may lead to false-positive results in the case of non–phosphaturic mesenchymal tumors producing trace amounts of FGF23 including fibrous dysplasia, chondromyxoid fibroma [29], and aneurysmal bone cyst [49].

## 9. Management

Resection of the tumor is the ultimate definitive treatment of tumor-induced osteomalacia. Maintaining a wide surgical margin cannot be emphasized enough to prevent the recurrence of the tumor which has been reported in several instances [27]. A 10 mm margin for extremity lesions and 5 mm for trunk lesions have been proposed. Since FGF23 has a short half-life of 45–58 minutes [50], a rapid resolution of symptoms upon surgical removal is expected.

Surgical cure is evidenced by normalization of the serum biochemical abnormalities including serum phosphate level. It is important to note that reversal of phosphate levels after surgical resection is required to confirm the diagnosis of TIO [13]. Late recurrence happens in a small percentage of patients [21, 27]; therefore, long-term follow-up is warranted after the surgery. Should metastasis occur, the lung is the most common site [21, 25–27]. In a review of 32 Japanese cases of PMTMCT, metastases were found in 4 patients (12.5%) on follow-up and 2 patients eventually died of the disease [25]. Radiofrequency ablation (RFA) has also received attention as a possible treatment modality which still needs further investigation [51]. Adjuvant radiotherapy, even though not well studied, has been recommended in cases of recurrence where repeat surgery would lead to significant disability such as losing a limb. Positive margins after surgery may also justify the use of postoperative radiotherapy.

If the tumor cannot be localized or surgically removed, medical therapy is recommended with phosphate supplementation and calcitriol [32]. The typical treatment regimen would be 15–60 mg/kg per day of elemental phosphate divided into 4–6 doses for 1–3 days. Calcitriol is given at 15–60 ng/kg per day, with a usual starting dose of 1.5 mg/day for an adult. Cinacalcet, an agonist of calcium-sensing receptors, has also led to promising outcomes, by lowering blood parathormone levels. Geller et al. [31] observed a significant bone-healing effect following treatment of two cases of TIO with 30 mg/day cinacalcet. Of note, despite the presence of somatostatin receptors on the cell surface of phosphaturic mesenchymal tumors, octreotide has not demonstrated significant clinical success [52].

Interestingly, the results of a phase I clinical trial on efficacy of a humanized anti-FGF23 antibody in adults with X-linked hypophosphatemic rickets (XLHR) indicated the safety and effectiveness of this novel treatment [53]. Given the similarity of pathogenesis, this antibody might be of therapeutic value for TIO as well.

## 10. Conclusion and Future Direction

In conclusion, TIO is a rare paraneoplastic syndrome caused by release of phosphate- and vitamin D-regulating mediators, most notably FGF23, from tumor cells. A distinct category of tumors, phosphaturic mesenchymal tumors, is responsible for majority of the cases. Excision of the culprit neoplasm is usually curative. Although these tumors are small and hard to locate on physical exam, a stepwise diagnostic approach involving functional and anatomical imaging studies and, if needed, selective venous sampling or aspiration of the lesion tends to be successful. Resection of the tumor is typically followed by a rapid decline in FGF23 levels and resolution of symptoms. Although characteristically benign, PMTs with aggressive features such as local invasion, metastasis, and late recurrence have also been described. When the tumor cannot be identified or resected, medical treatment with phosphate supplements, calcitriol, and cinacalcet is recommended.

Various aspects of TIO, ranging from pathogenesis to treatment, are yet to be understood. In terms of pathogenesis, the phosphaturic role of FGF7, which has recently been proposed, needs to be further elaborated in the future studies. From a diagnostic point of view, comparison between emerging modalities including somatostatin receptor PET tracers and more traditional imaging techniques such as MRI and PET/CT warrants further investigation. Finally, humanized anti-FGF23 antibody as a potential medical treatment for TIO is an area that is certainly worth exploring.

## Figures and Tables

**Figure 6:**
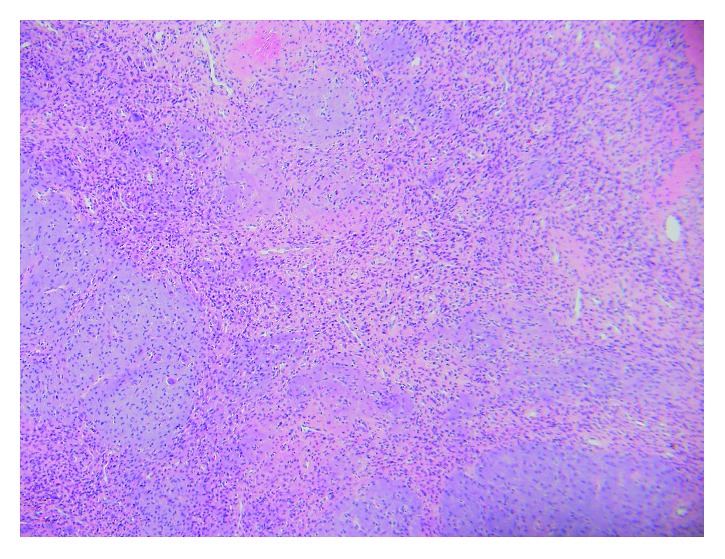
Low-power microscopic image (10x) showing a tumor consisting of prominent small vessels, myxoid stroma, and scattered osteoclast-type giant cells.

**Figure 7:**
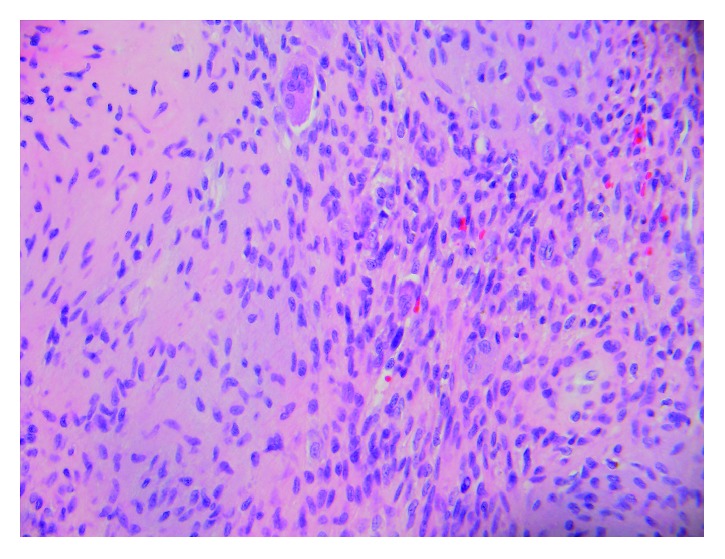
High-power microscopic image (40x) showing a tumor consisting of bland spindle cells sitting in slightly myxoid stroma with an osteoclast-type giant cell.

**Table 1:** Differential diagnoses of hypophosphatemia.

Decreased absorption	Increased excretion	Intercellular shifts
Poor dietary intake	Hyperparathyroidism: primary and secondary	Insulin effect, i.e., during refeeding syndrome
Inhibition of absorption due to medications including phosphate binders, anticonvulsants, antacids, etc.	Hereditary hypophosphatemic rickets: XLHR (mutations in PHEX gene), ADHR (mutations in FGF23 gene), ARHR (mutations in DMP1 gene), HHRH (mutations in sodium phosphate transporter 2c)	Respiratory alkalosis
Malabsorption syndromes, i.e., celiac disease, Crohn's, nontropical sprue, etc.	Fanconi syndrome: inherited versus acquired, i.e., heavy metal induced, chemotherapeutic agents, monoclonal gammopathies, etc.	Hungry bone syndrome
Vitamin D deficiency or resistance: dietary, lack of sunlight, excess fluoride, etc.	Tumor-induced osteomalacia	Increased metabolism: blast crisis, thyrotoxicosis

FGF23, fibroblast growth factor 23; XLHR, X-linked hypophosphatemic rickets; ADHR, autosomal dominant hypophosphatemic rickets; ARHR, autosomal recessive hypophosphatemic rickets; HHRH, hereditary hypophosphatemic rickets with hypercalciuria.

**Table 2:** Laboratory tests recommended in cases of suspected TIO.

Serum phosphorus
Serum calcium
25-Hydroxyvitamin D
1,25-Dihydroxyvitamin D
Parathyroid hormone
Alkaline phosphatase (bone-specific form if available)
24-hour urine collection for phosphorus, calcium, and creatinine
Fibroblast growth factor 23
Serum protein electrophoresis and immunofixation
Urine monoclonal proteins, kappa and lambda chains
